# Interaction Between Non-Coding RNAs and Interferons: With an Especial Focus on Type I Interferons

**DOI:** 10.3389/fimmu.2022.877243

**Published:** 2022-04-27

**Authors:** Soudeh Ghafouri-Fard, Yadollah Poornajaf, Farzaneh Dashti, Bashdar Mahmud Hussen, Mohammad Taheri, Elena Jamali

**Affiliations:** ^1^Department of Medical Genetics, School of Medicine, Shahid Beheshti University of Medical Sciences, Tehran, Iran; ^2^Faculty of Medicine, Birjand University of Medical Sciences, Birjand, Iran; ^3^Department of Pharmacognosy, College of Pharmacy, Hawler Medical University, Erbil, Iraq; ^4^Center of Research and Strategic Studies, Lebanese French University, Erbil, Iraq; ^5^Urology and Nephrology Research Center, Shahid Beheshti University of Medical Sciences, Tehran, Iran; ^6^Institute of Human Genetics, Jena University Hospital, Jena, Germany; ^7^Skull Base Research Center, Loghman Hakim Hospital, Shahid Beheshti University of Medical Sciences, Tehran, Iran; ^8^Department of Pathology, Loghman Hakim Hospital, Shahid Beheshti University of Medical Sciences, Tehran, Iran

**Keywords:** lncRNA, miRNA, interferon, expression, biomarker

## Abstract

Interferons (IFNs) are a group of cellular proteins with critical roles in the regulation of immune responses in the course of microbial infections. Moreover, expressions of IFNs are dysregulated in autoimmune disorders. IFNs are also a part of immune responses in malignant conditions. The expression of these proteins and activities of related signaling can be influenced by a number of non-coding RNAs. IFN regulatory factors (IRFs) are the most investigated molecules in the field of effects of non-coding RNAs on IFN signaling. These interactions have been best assessed in the context of cancer, revealing the importance of immune function in the pathoetiology of cancer. In addition, IFN-related non-coding RNAs may contribute to the pathogenesis of neuropsychiatric conditions, systemic sclerosis, Newcastle disease, Sjögren’s syndrome, traumatic brain injury, lupus nephritis, systemic lupus erythematosus, diabetes mellitus, and myocardial ischemia/reperfusion injury. In the current review, we describe the role of microRNAs and long non-coding RNAs in the regulation of IFN signaling.

## Introduction

Being firstly recognized as antiviral factors that interfere with viral replication (1), interferons (IFNs) are a group of cellular proteins classified in three families (2). Thirteen IFN-α variants, a single IFN-β and numerous other IFNs (IFN-ϵ, -k, -ω, and -δ) are classified as Type-I IFNs (3, 4). Type II IFN family only includes IFN-γ (5), a protein that potentiates proinflammatory signals through priming macrophages for antimicrobial functions and induction of nitric oxide synthesis and inhibition of the activity of NLRP3 inflammasome (6, 7).

Secretion of IFNs from infected cells can lead to induction of innate immune response resulting in cytokine release and induction of function of natural killer cells and antigen presentation (3, 8). These proteins have critical roles in the regulation of immune responses in the course of microbial infections. Moreover, expressions of IFNs are dysregulated in autoimmune disorders. Based on these roles, the identification of cellular mechanisms of regulation of IFNs has practical significance. Regulation of IFNs expressions is accomplished by different mechanisms, including of binding of regulatory molecules to their 3′ untranslated regions (3′ UTRs). This region contains both AU-rich elements (AREs) and microRNA (miRNA) recognition elements (2). RNA-binding proteins can target AREs and either induce mRNA degradation or stabilize mRNA. Meanwhile, the binding of miRNAs with miRNA recognition elements is involved in the regulation of mRNA translation *via* the miRNA-induced silencing complex (2). In the current review, we describe the role of miRNAs and long non-coding RNAs (lncRNAs) in the regulation of IFNs.

### Interactions Between miRNAs and IFNs

miR-301a has been found to contain a binding site in the 3′-UTR of the Interferon regulatory factor 1 (*IRF-1*) gene. Through the modulation of the expression of this gene, this miRNA participates in the proliferation of hepatocellular carcinoma cells. Expression of miR-301a has been increased in primary hepatocellular carcinoma tumors and cell lines, parallel with down-regulation of IRF-1. *In vitro* studies have shown the role of chronic hypoxia in the induction of miR-301a and down-regulation of IRF-1. Moreover, suppression of miR-301a induces cell apoptosis and reduces cell proliferation. Taken together, the regulation of miR-301a on IRF-1 expression is implicated in the pathogenesis of hepatocellular carcinoma (9). Another study in this malignancy has shown that IRF-1 can induce the expression of miR-195 to inhibit CHK1 expression. Up-regulation of IRF-1 or down-regulation of CHK1 induces cell apoptosis and increases PD-L1 expression in hepatocellular carcinoma cells (10).

In lung cancer cells, miR-19 has been shown to influence the expression of IFN-induced genes and MHC class I, signifying the impact of miR-19 in connecting inflammation and carcinogenesis (11). The IRF2-targeting miRNA miR-1290 has also been shown to be up-regulated in lung cancer. Over-expression of miR-1290 has been correlated with lymph node metastasis and advanced clinical stage. miR-1290 could enhance cell proliferation, colony formation, and invasive abilities in lung cancer cells. This miRNA could also promote the expression of cell proliferation-related proteins CDK2 and CDK4 and induce epithelial-mesenchymal transition (EMT) (12). Another study in lung cancer samples has shown up-regulation of IRF6 and down-regulation of miR-320, a miRNA that targets IRF6. IRF6 siRNA or miR-320 mimics could inhibit the growth and migration of lung cancer cells. Taken together, the miR-320/IRF6 axis has been suggested as a molecular axis involved in the pathogenesis of lung cancer (13).

Experiments in squamous cell carcinoma samples and cell liens have shown up-regulation of IRF2-targeting miRNA miR-664. This miRNA has been found to increase tumorigenic behaviors of cells both *in vitro* and *in vivo* (14).

Participation of IFN-related miRNAs has also been assessed in the pathoetiology of non-malignant conditions. For instance, the IRF2 targeting miRNA miR-221-3p has been found to be over-expressed in patients with the major depressive disorder compared with normal persons. Notably, serum miR-221-3p levels have been positively correlated with the level of depression. Mechanistically, miR-221-3p can enhance the expression of IFN-α in astrocytes through targeting IRF2. In fact, this miRNA participates in the induction of anti-neuroinflammatory signals by ketamine and paroxetine through the IRF2/IFN-α axis (15).

miR-126 and miR-139-5p are two miRNAs that participate in the dysregulation of plasmacytoid dendritic cells in systemic sclerosis. Expressions of these miRNAs have been correlated with the expression of type I IFN-responsive genes. TLR9 stimulation of plasmacytoid dendritic cells has induced expressions of miR-126 and miR-139-5p in cultures of normal cells as well as those obtained from patients with systemic sclerosis. USP24 has been identified as a target of miR-139-5p (16). **Table 1** summarizes the results of investigations that assessed the effects of miRNAs on IFN signaling.

**Table 1:** The effects of miRNAs on IFN signaling (ANT, adjacent normal tissue).

Type of diseases	miRNA	Sample	Cell Line	Target, Pathway	Discussion	Ref
Nasopharyngeal carcinoma	miR-9	–	CNE2, 5–8F	IFI44L, PSMB8, IRF5, PSMB10, IFI27, IFIT2, TRAIL, IFIT1	miR-9 modulates levels of IFN-induced genes and MHC class I.	(17)
Hepatocellular Carcinoma (HCC)	miR-301a (Up)	20 pairs of HCC and ANTs	Huh7, Hep3B, HepG2, Hepa1-6	IRF-1, Caspase-3	miR-301a *via* down-regulating IRF-1 could induce HCC.	(9)
HCC	miR-195 (–)	30 pairs of HCC and ANTs; WTB6 mice	Hepa1-6, Huh-7, Hep3B, HepG2	IRF-1, IFN-γ, CHK1, STAT3	IRF-1 *via* modulating miR-195 by down-regulating CHK1 could up-regulate apoptosis in HCC.	(10)
HCC	miR-146a (Up)	–	PLC/PRF/5	INF-α, SMAD4, STAT1/2	miR-146a could suppress the sensitivity to INF-α in HCC cells.	(18)
Lung Cancer (LC)	miR-19 (–)	–	CNE2, HONE1, A549, HCC827	IRF-1/7/9, IFI-6/27/35, HLA-B/F/G	miR-19 *via* regulating the expression of interferon could affect the expression of IFN-induced genes and MHC class I in human lung cancer cells.	(11)
Non-Small Cell Lung Cancer (NSCLC)	miR-1290 (Up)	41 pairs of NSCLC and ANTs	A549, H1299, SPC-A1, H1970, H460, BEAS-2B	IRF-2, CK2/4, E/N-cadherin	Overexpression of miR-1290 by targeting IRF2 could contribute to cell proliferation and invasion of NSCLC.	(12)
NSCLC	miR-320 (Down)	21 pairs of NSCLC and ANTs	A549, NCI-H2170	IRF-6	miR-320 *via* targeting IRF-6 could affect pathogenesis of NSCLC.	(13)
Cutaneous Squamous Cell Carcinoma (cSCC)	miR-664 (Up)	Athymic nude mice	HSC-1, A431, HSC-5, HaCaT	IRF-2	miR-664 *via* suppressing IRF-2 could function as an oncogene in cSCC.	(14)
Cervical Cancer (CC)	miR-587 (Up)	41 pairs of CC and ANTs, nude mice	Ect1/E6E7, HeLa, SiHa, CaSki, C-33A	IRF-6, Cyclin-D1, CDK4	miR-587 by repressing IRF6 could promote CC.	(19)
Gastric Cancer (GC)	miR-19a, miR-18a (–)	20 pairs of GC tissues and ANTs; BALB/c nude mice	MKN45, AGS, SGC7901, GES1	IFN-γ, IRF-1, Axin2, SMAD2, Wnt/β-catenin	IRF-6 by regulating MIR17HG-miR-18a/19a axis *via* Wnt/β-catenin signaling could promote GC metastasis.	(20)
Glioblastoma (GBM)	miR-203a (Down)	NSG mice	MT330, SJG2	IFN-α, IFN-β, IFN-λ1, IFI-1/6, IFT20, p65, NF-κB, STAT1-3	miR-203a *via* an ATM-dependent interferon response pathway could suppress GBM.	(21)
Osteosarcoma	miR-4295 (Up)	15 pairs of OS and ANTs	MG-63, Saos-2, hMSC	IRF-1	miR-4295 *via* targeting IRF1 could promote cell proliferation, migration and invasion.	(22)
Systemic Lupus Erythematosus (SLE)	miR-146	WBCs from patients with SLE	THP-1 cells		Type I IFN inhibits miR-146a maturation *via* increasing expression of MCPIP1.	(23)
Major Depressive Disorder (MDD)	miR-221-3p (Up)	(n=64) perioperative patients	Astrocytes	IRF-2, IFN-α, NF-κB	miR-221-3p *via* targeting IRF2 could up-regulate IFN-α expression in MDD patients.	(15)
Systemic Sclerosis	miR-126, miR-139-5p (Up)	Blood samples of SS patients (n=72) and healthy control (n=26)	pDCs	IFI-6, IFIT1, CXCL10, USP24, TLR-7/8/9	miR-126 and miR-139-5p *via* TLR9-mediated response and IFN signaling could regulate the activation of plasmacytoid dendritic cells.	(16)
Newcastle Disease (ND)	gga-miR-455-5p (Down)	–	293T, BHK-21	IFN-I, SOCS3	gga-miR-455-5p *via* targeting cellular suppressors of SOCS3 could suppress ND virus replication.	(24)
Sjögren’s Syndrome (SS)	miR-1248 (–)	–	phSG	IFN-β, IRF-1/9, IFIT1, IFI-6/44, IFIH1, MX1, JAK-1/2, STAT-1/2/3	miR-1248 could activate IFN-β *via* the direct association with both AGO2 and RIG-I.	(25)
Traumatic Brain Injury (TBI)	miR-155 (-)	C57BL/6 mice		IFN-I, IFN-α2/4/5, IL-6, IFN-β1, IRF-1, TNF-α, SOCS1C	Up-regulation of miR-155 after brain injury promotes IFN-I to exert a neuroprotective function.	(26)
Edwardsiella Tarda TX1 (E. tarda TX1)	pol-miR-194a (Up)	Fish	FG-9307, 293T	IFN-I, IRF-7	pol-miR-194a *via* targeting IRF7 could participate in the regulation of flounder immune response and microbial infection.	(27)
–	miR-17 (-)	C57BL/6J mice	VSMCs, RAVSMCs	IRF-9	miR-17 knockdown *via* up-regulating IRF-9 expression could promote vascular smooth muscle cell phenotypic modulation.	(28)
–	miR-155 (-)	–	EPC, BHK-21	IFN-I, PIAS4a	Overexpression of miR-155 *via* targeting IFN-I could contribute to antiviral response in EPC.	(29)
–	miR-181a, miR-30a (-)	–	U937, 293T, monocytes, MDM, MDDC	IFN-I/II, IFN-α, IFN-β, IFN-γ, ERK, STAT-1	Interferons *via* down-regulating miR-181a and miR-30a could induce the expression of SAMHD1 in monocytes.	(30)
–	miR-155, miR-155* (–)	–	HeLa, PDC	IFN-I, IFN-α, IFN-β, NF-κB, PI3K, AKT, p38	miR-155 in cooperation with its star-form partner miR-155* could regulate IFN-I production.	(31)
–	Bta-miR-204 (Down)	bEEC	–	IFN-τ, BoLA, PD-L1/2	IFN-τ by down-regulating bta-miR-204 could enhance the expression and function of BoLA.	(32)
–	miR-30c-5p (–)		Vero E6,	IFN-I/III, IFN-λ, IFIT1, ISG-15, SOCS-1	The coronavirus PEDV *via* the miR-30c-5p/SOCS1 axis could evade type III interferon response.	(33)
–	miR-744 (–)	–	RMCs	PTP1B, INF-I, CCL2/5, CXCL10, IL6, ERK, p38, MX1, IFIT3, TYK2, STAT1/3, JAK1, NF-κB	miR-744 by targeting PTP1B could enhance the INF-I signaling pathway in primary human renal mesangial cells (RMCs).	(34)
–	miR-155 (Up)	C57BL/6 mice	293T, RAW264.7, BMMs	SOCS1, IFN-β, MITF, TRAP	INF-β-induced miR-155 by targeting SOCS1 and MITF could inhibit osteoclast differentiation.	(35)
–	miR-221 (–)	–	H69, HIBEpiC	ICAM-1, IFN-γ, PRRSV, p65	miR-221 *via* targeting ICAM-1 translation regulating IFN-γ could induce ICAM-1 expression in human cholangiocytes.	(36)

miR-26a is an example of miRNAs that participate in the regulation of host immune responses during viral infections. Expression of this miRNA is increased upon infection with Feline Herpes Virus 1 (FHV-1). This virus could induce the expression of miR-26a through a cGAS-dependent route since down-regulation of cellular cGAS could result in blockage of poly (dA:dT) or FHV-1-induced expression of miR-26a. Functional studies have shown the impact of miR-26a in the induction of STAT1 phosphorylation and enhancement of type I IFN signals, which inhibit viral replication. In fact, miR-26a directly targets SOCS5 mRNA. SOCS5 silencing has led to an increase in STAT1 phosphorylation and induction of antiviral responses mediated by type I IFNs (37).

**Figure 1:**
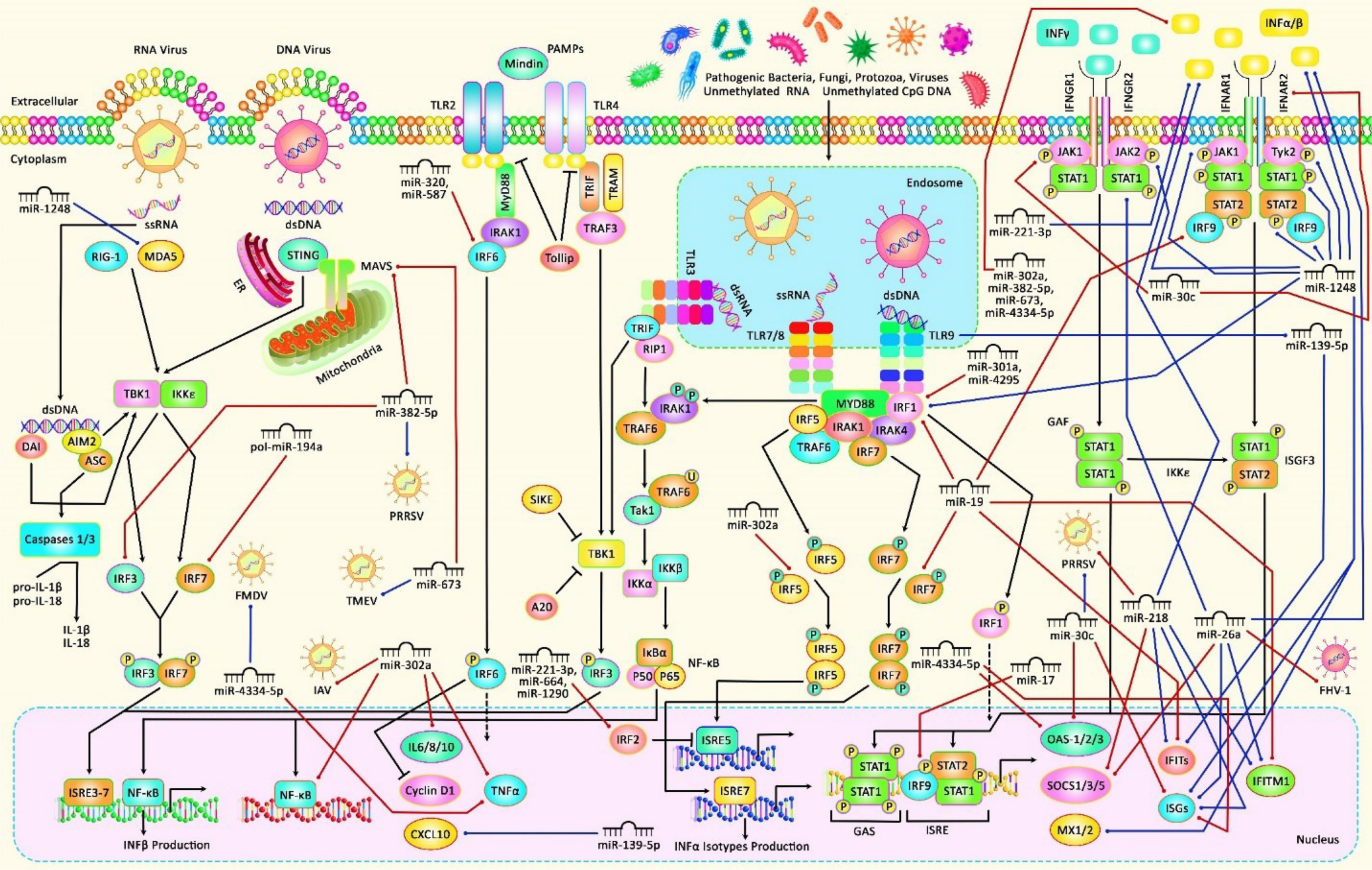
A schematic diagram of the interaction between several miRNAs and interferons in causing various human diseases. Mounting evidence has demonstrated that miRNAs could have an important contribution to the regulation of expression of IFN-induced genes. Aberrant expression of such ncRNAs could lead to various human diseases such as major depressive disorder, Sjögren’s Syndrome, Systemic Sclerosis as well as different kinds of cancers. As an illustration, a recent study has detected that overexpression of miR-301a could promote hepatocellular carcinoma *via* directly targeting IRF1 (9). Moreover, another research has figured out that miR-587 could play a key role in the progression of cervical cancer by down-regulating the expression of IRF6 (19). In addition, another finding has denoted that miR-1248 *via* activating the expression levels of IFN-β, IRF1/9, MX1, JAK-1/2, STAT-1/2, TYK2 as well as direct association with both AGO2 and RIG-I could have a crucial role in Sjögren’s syndrome (25). Furthermore, miR-26a could suppress feline herpesvirus 1 (FHV-1) replication *via* targeting SOCS5 and up-regulating the expression levels of IFN-α, IFN-β, ISG-15, STAT-1, and IFITM1 in type I IFN signaling (37). Blue lines indicate the positive regulatory effect among miRNAs and their targets, and crimson lines depict negative effects among them. All information regarding the role of these miRNAs in the modulation of the IFN signaling cascade in various types of human diseases and cancers can be seen in **Tables 1** and **2**.

Another study has shown a time-dependent down-regulation of miR-155 upon infection with the dengue virus. Exogenous up-regulation of this miRNA could limit replication of the dengue virus *in vitro*, indicating that down-regulation of miR-155 has a beneficial effect for replication of this virus. The results of *in vivo* experiments have also confirmed the impact of miR-155 in protection against the life-threatening effect of dengue virus infection. This activity of miR-155 has been shown to be exerted through targeting Bach1, and subsequent activation of the HO-1-mediated suppression of NS2B/NS3 protease activity of dengue virus. Taken together, modulation of miR-155 expression has been suggested as a therapeutic option for the management of dengue virus infection (38). miR-218 is another miRNA that can regulate host responses to viral infections since its down-regulation by porcine reproductive and respiratory syndrome virus can facilitate replication of this virus through suppression of type I IFN responses (39). **Table 2** shows the effects of miRNA on IFN signaling in the context of viral infections. **Figure 1** illustrates the aberrant expression of various miRNAs, which adversely affect the IFN signaling pathway triggering several kinds of human diseases and malignancies as well as their role in the context of viral infections.

**Table 2:** The effects of miRNA on IFN signaling in the context of viral infections.

Virus	miRNA	Sample	Cell Line	Target	Discussion	Ref
Infectious stress	miR-22	Mir22-KO mice	–	–	miR-22 enhances the IFN response to viral infections.	(40)
Infection with influenza virus	miR-144	Wild-type mice	–	TRAF6-IRF7	miR-144 diminishes host responses to the influenza virus.	(41)
Feline Herpes Virus 1(FHV-1)	miR-26a (Up)	–	F81, 293T	IFN-α, IFN-β, ISG-15, SOCS5, STAT-1	miR-26a by targeting SOCS5 and promoting Type I IFN signals could inhibit FHV-1 replication.	(37)
Human Herpes Simplex Virus Type 1 (HSV-1)	miR-23a (–)	–	HeLa	IRF-1, RSAD2, EGFP, Myc	miR-23a *via* suppression of IRF-1 could facilitate the replication of HSV-1.	(42)
Dengue Virus (DENV)	miR-155 (Down)	Breeder ICR mice	Huh-7	HO-1, IFN-α-2/5/17, BACH1, Nrf2, OAS-1/2/3	miR-155 by inducing HO-1-mediated antiviral interferon responses could inhibit DENV replication.	(38)
Porcine Reproductive & Respiratory Syndrome Virus (PRRSV)	miR-218, miR339-5p, miR-99b, miR-365-5p, miR-378, miR-345, miR-27b-3p	SPF pig	PAMs, marc-145, Vero-E6, ST, 293T	IFN-I, IFN-β, SOCS3	Downregulation of miR-218 by PRRSV could facilitate viral replication *via* repressing of type I IFN responses.	(39)
PRRSV	miR-30c (–)	–	PAM, Marc-145, U4A	IFN-I, IFNAR-2, ISG-15, OAS-1, JAK-1	miR-30c *via* targeting IFNAR-2 could promote type 2 PRRSV infection.	(43)
PRRSV	miR-382-5p (Up)	–	MARC-145, 293T, BHK-21	IFN-I, IFN-β, HSP60, MAVS, IRF-3, TBK1	miR-382-5p by negatively regulating the induction of IFN-I could promote PRRSV replication.	(44)
PRRSV2	miR-541-3p (Up)		MARC-145, MA-104, 293T	IRF7,	miR-541-3p *via* IRF7 could promote the replication of PRRSV2.	(45)
Influenza A virus, TMEV	miR-673 (–)	Dgcr8^-/-^ mouse, Dicer^+/+^ mouse	NIH3T3, ESC	IFN-β1, MAVS	During pluripotency, an interaction between MAVS (mitochondrial antiviral signaling protein) and miR-673 could act as a switch to suppress the antiviral IFN.	(46)
Influenza Virus A/WSN/33 (H1N1)	miR-302a (–)	C57BL/6 mice	A549, THP-1, 293T, MLE-12, H9	IFN-β, TNF-α, IRF-5, CCL-2/5, IL-6/8, M1, NP, NF-κB	miR-302a *via* targeting IRF5 expression and cytokine storm induction could suppress IAV.	(47)
H1N1	miR-93 (Down)	C57BL/6 mice	AT2, MLE12, A549, 293T, Murine T-cells, Murine B-cells, B-cells, NK cells	IFN-I, IFN-β, IRF-3, IL-6/8/10, NF-κB, ISG15, OAS1, RIG-I, p38/65, ERK, JAK-1/2	Inhibition of miR-93 by up-regulating JAK-1 could promote interferon effector signaling to suppress influenza A infection.	(48)
Influenza A virus (IAV) H5N1	miR-21-3p (Down)	26 H5N1-infected patients serum samples and 13 serum samples from normal persons	A549	IFN-I, FGF2, IFN-β, IFN-α, MxA, OAS	miR-21-3p by refraining IFN-I response could modulate FGF2 to facilitate influenza A virus H5N1 replication.	(49)
Foot & Mouth Disease Virus (FMDV)	miR-103, miR-107 (Down)	20 pairs of blood samples from patients with enterovirus 71 (EV71) and normal blood samples	VERO, RD	IFN-I, IFN-α, IFN-β, SOCS3, STAT3	miR-103/miR-107 by regulating SOCS3/STAT3 pathway could inhibit EV71 replication and facilitate IFN-I response.	(50)
FMDV	miR-4334-5p (Up)	–	PK-15, BHK-21	IFN-β, TNF-α, OAS, ISG54, ID1, VP1	miR-4334-5p by suppressing IFN pathways *via* direct targeting ID1 could facilitate FMDV propagation.	(51)
HIV-1	miR-128 (–)	–	HeLa, 293T, THP-1, Jurkat	INF I, IFN-α, TNPO3,	IFN-I *via* enhancing miR-128 by targeting TNPO3 mRNA could modulate HIV-1 Replication.	(52)
Infectious Bursal Disease Virus (IBDV)	gga-miR-27b-3p (Up)	–	DF-1	IFN-I, IFN-β, IRF3, NF-κB, SOCS3, SOC6, STAT-1	gga-miR-27b-3p *via* targeting cellular suppressors of SOCS3 and SOCS6 could enhance type I IFN signals and inhibit replication of IBDV.	(53)
IBDV	gga-miR-155 (Up)	–	DF-1	TANK, SOCS1, IFN-I, chIRF3	gga-miR-155 *via* targeting SOCS1, and TANK could enhance IFN-I and suppress IBDV.	(54)
IBDV	gga-miR-9* (Up)	–	DF-1	IRF-2, INF-β	gga-miR-9* by targeting IRF-2 to promote IBDV replication could inhibit IFN production in antiviral innate immunity.	(55)
Hepatitis C Virus (HCV)	miR-122 (–)	–	Huh7	INF-α, INF-β, EGFP, SOCS1	miR-122 *via* blocking suppressor of SOCS1 could modulate INF-I expression.	(56)
Human Papillomavirus 16 (HPV16)	miR-122 (–)	–	SiHa, CaSki, C33A	OAS-1, MxA, pmCherry-E6, IFN-α, IFN-β, STAT1, SOCS1	miR-122 *via* blocking suppressor of cytokine signaling 1 in SiHa cells could inhibit HPV E6 gene and enhance interferon signaling.	(57)
Human Cytomegalovirus (HCMV)	Hcmv-miR-UL112 (–)	–	PBMCs, K562	TNF-I, IFNAR, CD107	Hcmv-miR-UL112 activity by inhibiting INF-I secretion could attenuate NK cells.	(58)

### Interactions Between lncRNAs and IFNs

LncRNAs are a group of regulatory non-coding RNAs that share several characteristics with mRNAs, but lacking open reading frames. They participate in epigenetic regulation of gene expression through modulation of histone or DNA marks as well as regulation of the stability of RNAs and interacting with regulatory proteins (59).

Several lines of evidence suggest that lncRNAs include an important subgroup of the IFN target genes. Additionally, the IFN response has been shown to be regulated by several lncRNAs encoded by host or pathogens (60). Kambara et al. have identified approximately 200 lncRNAs whose expressions are induced by IFN in primary human hepatocytes (61). Notably, among them has been lncRNA-CMPK2/NRIR which has exhibited an intense induction after IFN stimulation in various human and mouse cells. This lncRNA is located near the protein-coding IFN-stimulated gene CMPK2. Expression of this lncRNA has been shown to be induced in a JAK-STAT-dependent manner. Silencing of lncRNA-CMPK2/NRIR has resulted in a significant decrease in HCV replication in IFN-induced hepatocytes, implying the role of this lncRNA in the modulation of antiviral effects of IFN (61).

NRAV is another lncRNA whose expression is regulated by IFNs. Microarray analyses in cells overexpressing this lncRNA has shown down-regulation of several ISGs. NRAV has been shown to be able to partially preclude IFN-induced expression of its target ISGs, possibly *via* affecting its transcription or through epigenetic mechanisms (62).

IFNG-antisense-1 (IFNG-AS1) is another lncRNA that participates in the regulation of IFN responses. This lncRNA is located downstream of the *IFNG* locus. Expression of IFNG-AS1 is strongly correlated with expression of IFNG (63, 64). CD4+ and CD8+ T cells as well as NK cells express this lncRNA. IFNG-AS1 expression by CD4+ T cells depends on two transcription factors being involved in Th1 polarization, namely STAT4 and TBX21 (65).

AFAP1-AS1, lncMX1-215, linc00513, BANCR, IFITM4P, LUCAT1, NEAT1, MALAT1, DANCR, NRIR, and FIRRE are among lncRNAs whose interactions with IFN signaling have been assessed (**Table 3**). For instance, the up-regulated lncRNA AFAP1-AS1 can participate in the invasiveness of lung cancer cells through increased expression of IRF7 and induction of RIG-I-like receptor signals (66). On the other hand, lncMX1-215 is an IFNα-induced lncRNA that can affect the immunosuppressive responses through interfering with H3K27 acetylation (67).

**Table 3:** Interactions between lncRNAs and IFNs.

Type of Diseases	LncRNAs	Sample	Cell Line	Target	Discussion	Ref
NSCLC	AFAP1-AS1 (Up)	NSCLC (n=165), banging lung tumor patients (n=118), health control (n=173)	A549, H1975, H1650, H1395 H12994	IRF-7, IFN-γ, RIG-I, Th1/2, IL-10/12, Bcl-2, TNF-α, NF-κB	AFAP1-AS1 *via* upregulating IRF-7 and the RIG-I-like receptor signaling could promote migration and invasion of NSCLC.	(66)
Head & Neck Squamous Cell Carcinoma (HNSCC)	lncMX1-215	70 HNSCC and 18 normal oral mucosa tissues from patients; BALB/c nude mice	HN4, HN6, HN30, Cal27, SCC4, SCC25, Detroit 562, 293T	IFN-α, H3K27ac, H3k18ac, H3K9ac, Caspase-3 PARP, Snail, STAT-1	IFN-α-induced lncMX1-215 by interfering with H3K27 acetylation could decrease immunosuppression in HNSCC.	(67)
Cryptosporidium Infection	NR_033736	BV2 mice,	IEC4.1, HCT-8, BV2, RAW264.7	IFN-α, IFN-β1, IFN-α12/13, IFN-I, ISGF-3, IFI-44, IFIT-1, OAS2/3, IRF-9, H3K4me3, STAT-2	NR_033736 *via* regulating IFN-I-mediated gene transcription could induce intestinal epithelial anti-cryptosporidium defense.	(68)
Lupus Nephritis (LN)	RP11-2B6.2 (UP)	22 LN kidney biopsies and 7 control samples, PBMC	HeLa, HK2	IFN-I, IFI27, IFIT-1/3, ISG, Mx2, OASL, ASO1, CXCL10, JAK1, STAT-1, SOCS1	RP112B6.2 *via* targeting the IFN-I by epigenetically inhibiting the expression of SOCS1 could aggravate symptoms of LN disease.	(69)
Systemic Lupus Erythematosus (SLE)	linc00513 (–)	139 SLE patients	Hela, THP-1, PBMCs	IFN-I, IRF-9, OAS-1/2/3, IFI-27/44/44L, ISG-15/20, IFIT-1/3, Mx1/2, XAF1, NF-kB, STAT-1/2	Overexpression of linc00513 *via* promoting IFN signaling could play a role in lupus pathogenesis.	(70)
Diabetes Mellitus Type 1	Lnc10 (–)	–	EndoC-βH1	IFN-I, IFN-γ, IFITM1, IL-1β, STAT-1	Overexpression of lnc10 *via* IFN-I could enhance the immune response in pancreatic β-cells.	(71)
Myocardial I/R Injury	BANCR (–)	–	iPS cell-derived cardiomyocytes	IFN-β, IFNAR-1, STAT-1/2	BANCR by targeting STAT-1 could promote IFN-β-induced cardiomyocyte apoptosis.	(72)
Infectious Bursal Disease Virus (IBDV)	loc107051710 (–)	–	DF-1	IRF-8, IFI-1/6, IFN-α, IFN-β, Mx1, IFIT-5, STAT-1/2	loc107051710 by regulating IRF-8 could promote the production of IFN-α and IFN-β, thereby modulating the antiviral activity of ISGs.	(73)
Influenza A Virus (IAV)	IVRPIE (Up)	–	A549, BEAS-2B, MDCK, BHK21	IFN-β1, ISG, IRF-1, IFIT-1/3, Mx1, ISG-15, IFI44L	IVRPIE *via* regulating IFN-β1 and ISG expression could promote host antiviral immune responses.	(74)
Influenza A Virus (IAV); H1N1, IAV-PR8, IAV- CA04	ISR (–)	C57BL/6 mice	A549, 293T, NIH/3T3, 4T1, MDCK	IFN-β, IFNAR-1, RIG-I, MxA, ISG-15, OAS2	ISR could be regulated by RIG-I-dependent signaling; during IAV infection, it could also govern IFN-β production and inhibit viral replication.	(75)
Influenza Virus A/WSN/33 (H1N1)	IFITM4P (–)	–	A549, 293T, K562, HeLa, MDCK, Huh7, Mcf7, HepG2	IFITM-1/2/3, miR-24, Mx1, RIG-I, p65, IL-6	IFITM4P by acting as a competing endogenous RNA could regulate host antiviral responses.	(76)
Influenza Virus A/WSN/33 (H1N1), Sendai Virus (SeV)	Lnc-MxA (–)	–	MDCK, 293T, A549,	IFN-β, RIG-I, MAVS, IRF-3, INFAR-1, p65, ISG-15, MxA	Lnc-MxA by forming RNA-DNA triplexes could inhibit β interferon transcription.	(77)
Herpes Simplex Virus 1 (HSV-1), Influenza A Virus (IAV), LPS	LUCAT1 (–)	PBMCs	THP-1, THP-1 KO, hMDDC	IFN-I, IFN-α, IFN-β, IRF-3, IFI1-6, ISG, TNF-α, Mx2, JAK-1/3, STAT1	LUCAT1 by interacting with STAT1 in the nucleus could limit the transcription of ISGs.	(78)
Severe Acute Respiratory Syndrome Coronavirus 2 (SARS-CoV-2)	RP1-20B21.4, RP11-329L6.1, RP11-498C9.3, NEAT1, MALAT1 (–)	Dataset	–	miR-122, miR-122-5p, IRF-9, IFIT-1/2/3, MX1, OAS2/3, IFNL-1 IFNG, JAK, STAT-1	The SARS-CoV-2 infection could lead to differential expression of lncRNAs. Also, IFN response is involved in SARS-CoV-2 infection.	(79)
HIV-1-BAL-HSA	NRIR, MIR3945HG, C8orf3, AC053503.1, AL359551.1 (–)f	–	CD14^+^ monocytes, MDMs	IFN-α, IFN-ϵ, IFN-γ, IFN-λ, Mx1, IFIT2	Interferons could mediate the Response of lncRNAs in macrophages in HIV.	(80)
Vesicular Stomatitis Virus (VSV), VSV-GFP	lncLrrc55-AS (–)	C57BL/6 mice	RAW264.7, NIH/3T3, 293T, MDCK, MLE12, 3LL, Hepa	IFN-α4, IFN-β, IRF-3, IFN-I, p65, p38, ERK, JAK, STAT-1	Interferon-inducible cytoplasmic lncLrrc55-AS by strengthening IRF3 phosphorylation could promote antiviral innate responses.	(81)
–	GRASLND, NEAT1 (–)	–	ASCs	IFN-II, IFN-α, IFN-β, IFN-γ, IRF-1/2/6, IFI-44/44L, IFNGR-1/2, STAT-1/2	GRASLND *via* suppressing the IFN-II pathway could enhance chondrogenesis.	(82)
–	BANCR (–)	–	ARPE-19,	IFN-γ, IL-1β, TNF-α, JAK, STAT-1	IFN-γ by activating the JAK-STAT1 pathway could upregulate the expression of BANCR in retinal pigment epithelial cells.	(83)

LncRNAs can also affect response to protozoan parasites such as cryptosporidium. NR_033736 is a novel lncRNA that has been found to be up-regulated in intestinal epithelial cells upon infection with this protozoon. This lncRNA can suppress transcription of type I IFN-controlled genes in host cells infected with this microorganism. Notably, type I IFN signaling can trigger the expression of NR_033736. In fact, NR_033736 participation in the negative feedback regulatory mechanism of type I IFN signaling results in fine-tuning of innate defense mechanism against microorganisms in the epithelial cells (68).

Investigations in the context of lupus nephritis have shown that RP112B6.2 *via* targeting the IFN-I by epigenetically inhibiting the expression of SOCS1 could aggravate symptoms of this disease (69). Linc00513 is another lncRNA that participates in the pathogenesis of lupus through promoting IFN signaling (70).

**Figure 2:**
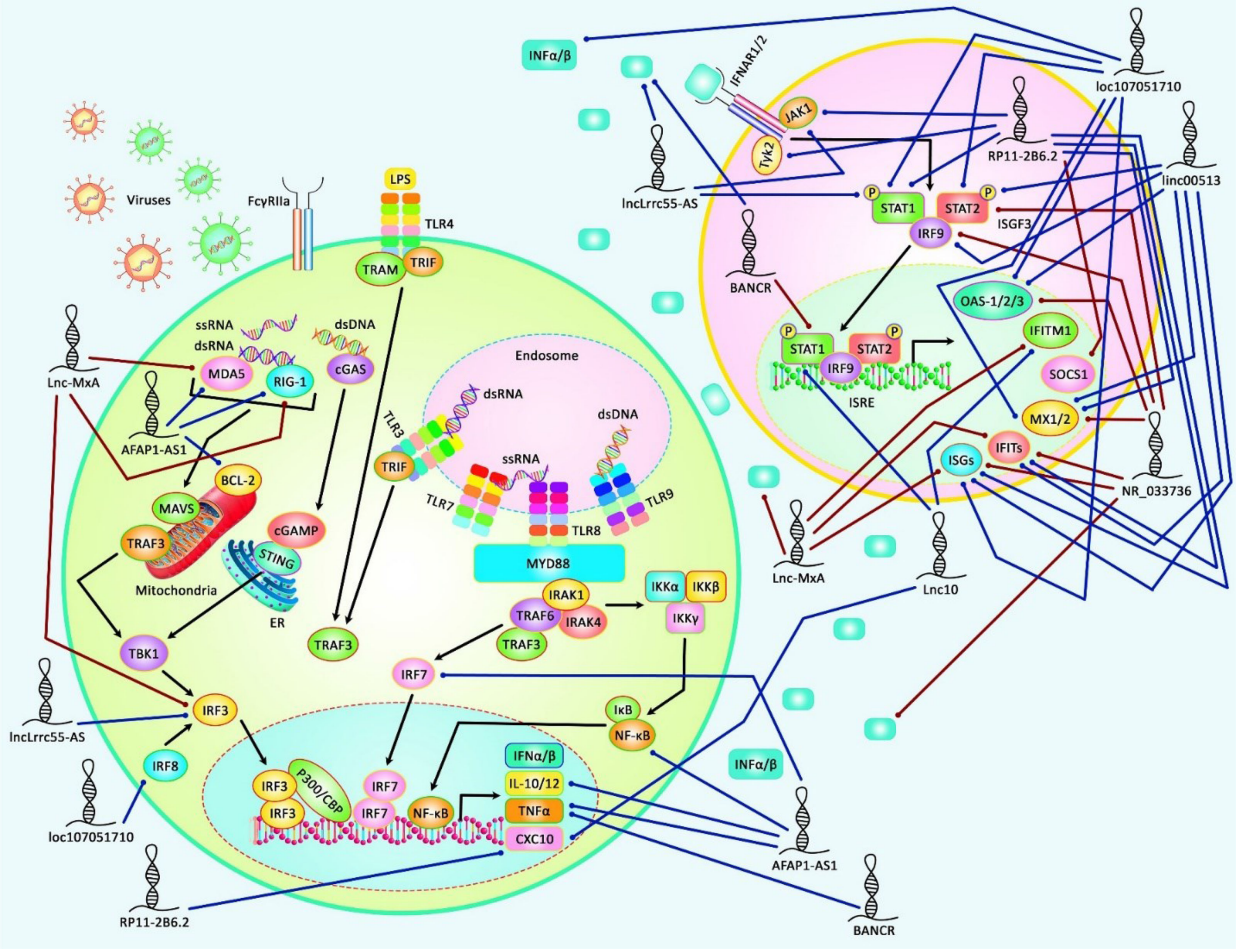
A schematic illustration of the role of several lncRNAs in regulating the IFN signaling pathway in several human diseases, including autoimmune conditions and viral infections. Accumulating evidence has illustrated that lncRNAs modulating IFN signaling cascade could participate in the pathogenesis of various kinds of human cancers as well as immune-related disorders. It has been reported that lncRNA RP11-2B6.2 could play an important role as a positive regulator of type I INF signaling pathway in Lupus Nephritis *via* up-regulating the expression levels of IFIT-1/3, ISG, Mx2, CXCL10, JAK1, STAT-1, TYK2, and decreasing SOCS1 expression (69). Moreover, another research has revealed that lncRNA loc107051710 could elevate the expression levels of IFN-α, IFN-β, Mx1, STAT-1/2, OAS *via* modulating IRF8, thereby enhancing the antiviral activity of ISGs to prevent infectious bursal disease virus (IBDV) infection (73). Blue lines indicate the positive regulatory effect among lncRNAs and their targets, and brown lines depict a negative one among them. All the information regarding the role of these lncRNAs involved in the modulation of the IFN signaling cascade in various types of immune deficiency diseases and cancers can be seen in **Table 3**.

The impact of lncRNAs on IFN signaling has also been assessed in the context of diabetes mellitus. Lnc10 contains a type I diabetes-associated single nucleotide polymorphism. This lncRNA can regulate the expression of the IRF7-driven inflammatory network regulating gene Ebi2 in immune cells. Expression of Lnc10 in pancreatic β-cells has been shown to be up-regulated by diabetogenic incitements, including pro-inflammatory cytokines and viral infections (71). **Figure 2** represents the role of several lncRNAs in various types of human cancers and immune-related disorders as well as their impact on viral infections *via* regulating the IFN signaling pathway.

## Discussion

Non-coding RNAs that regulate IFN signaling have been shown to participate in the pathogenesis of different types of cancer as well as immune-related disorders. IRFs are the most investigated molecules in the field of effects of non-coding RNAs on IFN signaling. For instance, IRF-1 has been shown to have functional interactions with miR-301a, miR-195, miR-19a, miR-18a, miR-4295, miR-124, and miR-155. Meanwhile, IRF-2 has interactions with miR-1290, miR-664, and miR-221-3p. Besides, IRF-6 interacts with miR-320, miR-587, miR-19, and miR-18a. These interactions have been best assessed in the context of cancer, revealing the importance of immune function in the pathoetiology of cancer.

In addition, IFN-related non-coding RNAs may contribute to the pathogenesis of neuropsychiatric conditions through modulation of immune responses in CNS-resident cells. Major depressive disorder is an example of these conditions in which the role of IRF-targeting miRNAs has been identified. Among non-malignant conditions, are systemic sclerosis, Newcastle disease, Sjögren’s syndrome, traumatic brain injury, lupus nephritis, systemic lupus erythematosus, diabetes mellitus, and myocardial ischemia/reperfusion injury have been found to be associated with dysregulation of IFN-related non-coding RNAs. These non-coding RNAs represent a novel group of biomarkers for these conditions since their expressions are dysregulated in the biofluids of patients with these disorders.

Consistent with the important role of IFN signaling in the response of the immune system to viral infections, non-coding RNAs that regulate these signals can also participate in the pathophysiology of these conditions. The interactions between non-coding RNAs and IFN signaling have been assessed in the context of SARS-CoV-2, HIV, and influenza infections. Particularly, some miRNAs have been reported to enhance antiviral responses through modulation of IFN signaling. Identification of the impact of these miRNAs in response to viral infections could facilitate the design of efficient therapeutic modalities for these disorders. The preliminary results of *in vitro* and *in vivo* studies have suggested modulation of expression of certain miRNAs as an efficient strategy for limiting viral infections.

Notably, single nucleotide polymorphisms in the seed region of IFN-interacting miRNAs can interfere with or induce their bindings with miRNA targets. These polymorphic regions can hypothetically affect IFN responses, thus participating in the pathogenesis of autoimmune disorders, malignancies, or viral infections. Identification of these variants within the human genome might facilitate the design of specific treatment modalities for these conditions in the context of personalized medicine.

Several dysregulated IFN-related miRNAs, particularly miR-9, miR-18, miR-301a, miR-195, miR-19, miR-1290, miR-320, miR-664, miR-587, miR-203a, and miR-4295 have been shown to participate in the pathogenesis of human cancers. These miRNAs represent appropriate targets for anti-cancer therapies since they can affect immune responses against cancer. Future studies are needed to evaluate the effects of these miRNAs-targeting therapies in xenograft models of cancer.

Taken together, non-coding RNAs that regulate IFN signaling can participate in a variety of malignant and non-malignant disorders, particularly those related to abnormal immune responses.

## Author Contributions

SG-F wrote the draft and revised it. MT designed and supervised the study. EJ, BH, YP and FD collected the data and designed the figures and tables. All the authors read and approved the submitted version.

## Conflict of Interest

The authors declare that the research was conducted in the absence of any commercial or financial relationships that could be construed as a potential conflict of interest.

## Publisher’s Note

All claims expressed in this article are solely those of the authors and do not necessarily represent those of their affiliated organizations, or those of the publisher, the editors and the reviewers. Any product that may be evaluated in this article, or claim that may be made by its manufacturer, is not guaranteed or endorsed by the publisher.

## Abbreviations

ESCs, Embryonic Stem Cells; FGF2, Growth Factor2; bEEC, Primary Bovine Endometrial Epithelial Cell; BoLA, Bovine Leukocyte Antigen; VSMCs, Vascular Smooth Muscle Cells; RAVSMCs, Rat Aortic VSMCs; RD, Rhabdomyosarcoma Cells; pDCs, Plasmacytoid Dendritic Cells; hMSC, Normal Human Mesenchymal Stem Cells; ATM, Ataxia-Telangiectasia Mutated; MDDCs, Monocyte-Derived Dendritic Cells; MDM, Monocyte-Derived Macrophage (MDMs); PBMC, Peripheral Blood Mononuclear Cell; GES1, Gastric Epithelial Cell Line; HSP60, Heat Shock Protein 60; MAVS, Mitochondrial Antiviral Signaling Protein; BHK, Baby Hamster Kidney; DENV, Dengue Virus; FHV-1, Feline Herpesvirus 1; CHK1, Checkpoint Kinase 1; INCR1, IFN-Stimulated Non-Coding RNA 1; ASCs, Adipose-Derived Stem Cells; MDCK, Madin-Darby Canine Kidney; mulNTEPI, Murine Intestinal Epithelial; BoLA, Bovine Leukocyte Antigen; PME-1, Phosphatase Methylesterase 1; PAMs, Porcine Alveolar Macrophages; VERO, African Green Monkey Kidney Cells; RD, Human Rhabdomyosarcoma Cells; EPC, Epithelioma Papulosum Cyprini; LPS, lipopolysacaridase; IRF-1, Interferon Regulatory Factor-1; ISGs, Interferon-Stimulated Genes; IFNAR, Heterodimeric Interferon Receptor; hMDDC, Primary Human Monocyte-Derived Dendritic Cells; MDMs, Monocyte-Derived Macrophages; ASCs, Adipose-Derived Stem Cells; LPS, Lipopolysaccharide; bEEC, Primary Bovine Endometrial Epithelial Cell; RIG-I, Retinoic Acid-Inducible Gene I; IFT, Intraflagellar Transport; IFIT1, Interferon Induced Protein with Tetratricopeptide Repeats 1; IFIH1, Interferon Induced with Helicase C Domain 1.

## Glossary

**Table:**

ESCs	Embryonic Stem Cells
FGF2	Growth Factor2
bEEC	Primary Bovine Endometrial Epithelial Cell
BoLA	Bovine Leukocyte Antigen
VSMCs	Vascular Smooth Muscle Cells
RAVSMCs	Rat Aortic VSMCs
RD	Rhabdomyosarcoma Cells
pDCs	Plasmacytoid Dendritic Cells
hMSC	Normal Human Mesenchymal Stem Cells
ATM	Ataxia-Telangiectasia Mutated
MDDCs	Monocyte-Derived Dendritic Cells
MDMs	MDM, Monocyte-Derived Macrophage
PBMC	Peripheral Blood Mononuclear Cell
GES1	Gastric Epithelial Cell Line
HSP60	Heat Shock Protein 60
MAVS	Mitochondrial Antiviral Signaling Protein
BHK	Baby Hamster Kidney
DENV	Dengue Virus
FHV-1	Feline Herpesvirus 1
CHK1	Checkpoint Kinase 1
INCR1	IFN-Stimulated Non-Coding RNA 1
ASCs	Adipose-Derived Stem Cells
MDCK	Madin-Darby Canine Kidney
mulNTEPI	Murine Intestinal Epithelial
BoLA	Bovine Leukocyte Antigen
PME-1	Phosphatase Methylesterase 1
PAMs	Porcine Alveolar Macrophages
VERO	African Green Monkey Kidney Cells
RD	Human Rhabdomyosarcoma Cells
EPC	Epithelioma Papulosum Cyprini
LPS	lipopolysacaridase
IRF-1	Interferon Regulatory Factor-1
ISGs	Interferon-Stimulated Genes
IFNAR	Heterodimeric Interferon Receptor
hMDDC	Primary Human Monocyte-Derived Dendritic Cells
MDMs	Monocyte-Derived Macrophages
ASCs	Adipose-Derived Stem Cells
LPS	Lipopolysaccharide
bEEC	Primary Bovine Endometrial Epithelial Cell
*RIG*-I	Retinoic Acid-Inducible Gene I
IFT	Intraflagellar Transport
IFIT1	Interferon Induced Protein with Tetratricopeptide Repeats 1
IFIH1	Interferon Induced with Helicase C Domain 1
